# Fructose impairs glucose-induced hepatic triglyceride synthesis

**DOI:** 10.1186/1476-511X-10-20

**Published:** 2011-01-24

**Authors:** Danshan Huang, Tania Dhawan, Stephen Young, William H Yong, Laszlo G Boros, Anthony P Heaney

**Affiliations:** 1 Department of Medicine, David Geffen School of Medicine at UCLA (Westwood Blvd.), Los Angeles (CA 90095), USA; 2 Department of Neurosurgery, David Geffen School of Medicine at UCLA, (Westwood Blvd.), Los Angeles (CA 90095), USA; 3 Department of Pathology, David Geffen School of Medicine at UCLA, (Westwood Blvd.), Los Angeles (CA 90095), USA; 4 SIDMAP LLC (2990 South Sepulveda Blvd.), Los Angeles (CA 90064), USA

## Abstract

Obesity, type 2 diabetes and hyperlipidemia frequently coexist and are associated with significantly increased morbidity and mortality. Consumption of refined carbohydrate and particularly fructose has increased significantly in recent years and has paralled the increased incidence of obesity and diabetes. Human and animal studies have demonstrated that high dietary fructose intake positively correlates with increased dyslipidemia, insulin resistance, and hypertension. Metabolism of fructose occurs primarily in the liver and high fructose flux leads to enhanced hepatic triglyceride accumulation (hepatic steatosis). This results in impaired glucose and lipid metabolism and increased proinflammatory cytokine expression. Here we demonstrate that fructose alters glucose-stimulated expression of activated acetyl CoA carboxylase (ACC), pSer hormone sensitive lipase (pSerHSL) and adipose triglyceride lipase (ATGL) in hepatic HepG2 or primary hepatic cell cultures *in vitro*. This was associated with increased de novo triglyceride synthesis *in vitro *and hepatic steatosis *in vivo *in fructose- versus glucose-fed and standard-diet fed mice. These studies provide novel insight into the mechanisms involved in fructose-mediated hepatic hypertriglyceridemia and identify fructose-uptake as a new potential therapeutic target for lipid-associated diseases.

## Introduction

Obesity, type 2 diabetes and hyperlipidemia frequently coexist and are associated with significantly increased morbidity and mortality [1]. A significant increase in total refined carbohydrate intake and particularly fructose has paralled recent increased incidence of obesity and diabetes. Metabolism of sugars and particularly fructose occurs primarily in the liver and high fructose flux leads to enhanced hepatic triglyceride accumulation resulting in impaired glucose and lipid metabolism and increased proinflammatory cytokine expression [2-6].

The role that triglycerides play as an independent risk factor for CAD is still not well defined but a substantial number of individuals who maintain total plasma cholesterol concentrations within acceptable values (< 5.17 mmol/L or 200 mg/dL) still develop CAD [7,8]. Some of these patients have low HDL -cholesterol concentrations (< 0.91 mmol/L or 35 mg/dL) [9,10] and a decline in HDL-cholesterol often accompanies an increase in VLDL triglyceride, reflecting an interchange of cholesterol esters from HDL for triglyceride from VLDL [11-16]. In addition to lipid abnormalities, hyperinsulinemia also plays an important role in CAD risk and triglyceride concentrations are highly correlated to plasma insulin response to carbohydrate [17-22].

Given that dietary fructose has been associated with hypertriglyceridemia and insulin resistance, primarily of hepatic origin, the aim of this study was to evaluate the effects of fructose on hepatic intracellular triglyceride metabolism and gain some insight into the mechanisms by which fructose enhances hepatic lipogenesis. Here we demonstrate that addition of fructose to human hepatic HepG2 cells and primary murine hepatic cell cultures incubated in physiologic and diabetic-range glucose concentrations *in vitro *disrupts normal glucose metabolism and leads to hepatic triglyceride accumulation in association with reduced hepatic expression of phosphorylated acetyl CoA carboxylase (pSer^479 ^ACC), phopshorylated hormone sensitive lipase (pSer^660 ^HSL) and total adipose triglyceride lipase (ATGL) in comparison to glucose alone. Additionally fructose-fed athymic Nu/Nu mice exhibited increased hepatic steatosis in association with reduced hepatic ATGL expression in comparison to glucose- and standard defined diet-fed mice.

## Materials and methods

### Cell culture and hepatocyte isolation

Human hepatoma HepG2 cells purchased from ATCC were grown at 37°C in 5% CO_2 _in DMEM medium with 10% FBS, 500 mM Hepes, glutamine, penicillin (100 U/ml) and streptomycin (100 ug/ml) (Invitrogen). Hepatic tissue was harvested from 3-mth old male C57/Bl6 mice and primary hepatic cell culture performed as previously reported [23]. At 30% confluence, HepG2 or primary hepatocyte cells were treated with glucose free DMEM with 5% FBS overnight and then incubated with glucose ( 5.5 and 11.1 mM) or fructose ( 0.55 mM) concentrations alone or fructose (0.55 mM) in combination with physiological glucose (5.5 mM) or high glucose (11.1 mM) for 72 hours. Tissue culture materials and other reagents were obtained from Sigma Chemical Co. (St. Louis, MO).

### Metabolomic Studies

75% confluent cultures of HepG2 cells (3 × 10^6^) were incubated for 72 h in 5.5 mM glucose, 11.1 mM glucose alone or 5.5 mM glucose in combination with 5.5 mM fructose, all of which contained a 10% solution of ^13^C tracer - [1,2-^13^C_2_] D-glucose (> 99% purity, and 99% isotope enrichment for each carbon position) (Cambridge Isotope Labs, Massachusetts) in T75 culture flasks. Following the glucose- or combination glucose/fructose treatment, culture medium was collected and cells were washed twice in PBS. Cell pellets were then harvested and palmitate, and oleate were extracted after saponification of cell pellets in 30% KOH and 100% ethanol using petroleum ether. Fatty acids were then converted to their methylated derivative using 0.5N methanolic-HCL and mass spectral data then obtained on a HP5973 mass selective detector connected to an HP6890 gas chromatograph. Palmitate and oleate were monitored at m/z 270 and m/z 264, respectively, with the enrichment of ^13^C labeled acetyl units to reflect synthesis, elongation and desaturation of the new lipid fraction as determined by mass isotopomer distribution analysis (MIDA) [24].

### Western blotting

Cell lysates or homogenized frozen hepatic tissue (100-120 mg) were solubilized in buffer. Lysates were cleared by centrifugation and total protein concentration determined using a BioRad DC kit. 30 ug of the proteins were separated by 10% SDS-PAGE and transferred to nitrocellulose membranes, followed by immunoblotting using specific primary and secondary antibodies and visualized using SuperSignal Chemiluminescence Assay kit (Pierce Inc., Rockville, MD). Antibodies to pSer^79 ^ACC, total ACC, pSer^660 ^HSL, total HSL and ATGL were from Cell Signaling Technology (Danvers, MA) and β-actin was from Santa Cruz Biotechnology (Santa Cruz, CA).

### Animals and Diet

All procedures were approved by the UCLA Animal Research Committee. Three-month old male athymic Nu/Nu mice purchased from Jackson Labs were housed individually in open-topped cages undera 12-hour light and 12-hour dark regimen and groups of six mice fed isocaloric (3675 kcal./kg) defined diets; standard (STD)diet (10% sucrose, 47% cornstarch), high glucose (HG, 60% glucose) or high fructose (HF, 60% fructose) diets [Dyets Inc, Pennsylvania, USA] for 12 weeks. Food intake and body weights were determined weekly. For intraperitoneal glucose tolerance tests (IPGT), mice were fasted overnight and retro-orbital blood glucose monitored at baseline (0), 15, 30, 60 and 120 minutes using a glucometer (Lifescan) after intraperitoneal glucose loading (1 g glucose per kg body weight) and area under the curve (AOC, mg/dl × min) for glucose levels during IPGT's was calculated for each group as previously described. All assays were carried out in triplicate and expressed asmean ± SEM. Insulin was measured in plasma samples by RIA (Linco, St. Charles, Missouri). Histological analysis was performed on paraffin-embedded hepatic tissues using consecutive 5-μm sections stained with hematoxylin and eosin. Briefly, 1/3 of the left posterior lobe of the liver was fixed in 10% neutral-buffered formalin for 24h, processed though graded alcohols and xylene and embedded in paraffin. 5-μm sections were deparaffinized and stained with hematoxylin and eosin (HE) using routine methods. Fat deposition was also analyzed in 8-μm frozen liver sections stained with 0.18% oil red O (Sigma-Aldrich) with 60% 2-propanol (Sigma-Aldrich) for 20 minutes at 37° and counterstained with hematoxylin. Fat accumulation stained with oil red O was quantified using an Image-Pro Plus Analyzer (Media Cybernetics, Inc., Bethesda, MD) by a pathologist (W. Yong). To quantify hepatic triglyceride content, liver tissues (100 mg) were homogenized in ice-cold 20 mmol/L Tris-HCl, 150 mmol/L NaCl, 2 mmol/L EDTA, and 1% Triton X-100, pH 7.5, triglycerides extracted with chloroform/methanol (2:1) and triglycerides quantitated using a commercially available kit (Infinity Triglycerides Stable Reagent, Thermo Electron) and correlated to total hepatic protein content. Oil Red O-staining was used to measure cellular neutral lipid droplet accumulation *in vitro *in the HepG2cells [25].

### Statistical analysis

Results are expressed as mean ± SEM (metabolomic experiments mean +/- SD) and analysis performed by ANOVA with Bonferroni comparison tests or nonparametric *t- *test (one tailed/unpaired) as indicated. *P *values < 0.05 were considered significant.

## Results

### Fructose increases *in vitro *hepatic triglyceride storage and secretion

**Figure 1:**
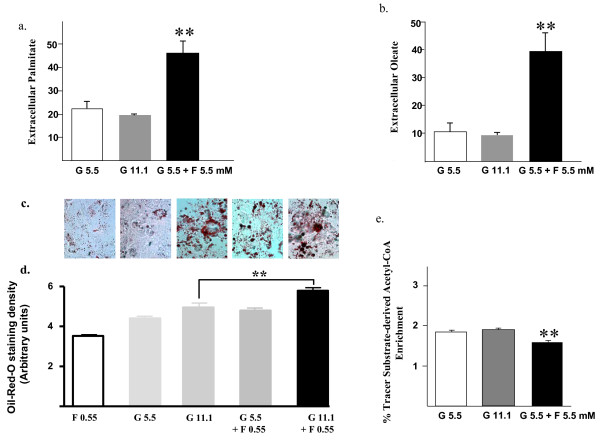
**Fructose increases glucose-stimulated triglyceride synthesis**. Metabolomic studies in HepG2 cells treated with either glucose (5.5 or 11 mM) alone or or admixed glucose (5.5 or 11.1 mM) and fructose (5.5 mM) concentrations depicting enrichment of ^13^C labeled acetyl units reflecting synthesis, elongation and desaturation of new lipid fractions as determined by mass isotopomer distribution analysis (MIDA) (a) extracellular palmitate and (b) oleate. Photomicrographs (c) and quantitation (d) of of oil-red-O staining in human HepG2 cells following incubation (72h) in fructose (0.55 mM) and glucose (5.5 & 11.1 mM) alone or admixed glucose (5.5 or 11.1 mM) and fructose (0.55 mM) concentrations. (e) Relative Acetyl CoA enrichment from the [1,2-^13^C_2_] D-glucose. G; glucose; F, fructose. *, p < 0.05; ** p < 0.01.

Following growth of HepG2 cells for 72 h in medium containing either 5.5 mM or 11.1 mM glucose alone or 5.5 mM glucose in combination with 5.5 mM fructose, all of which contained a 10% solution of ^13^C tracer - [1,2-^13^C_2_] D-glucose, we analyzed cell media and pellets by GC-mass spectroscopy to quantitate intra- and extra-cellular plamitate and oleate levels to examine the effects of fructose on hepatic triglyceride metabolism *in vitro*. As depicted in figure 1, extracellular palmitate levels were 2-fold higher when fructose was added to HepG2 cells incubated in physiologic and diabetic range glucose concentrations compared to glucose alone (figure 1a) (Mean ± SD extracellular palmitate: G 5.5 mM 23 ± 3; G 11.1 mM, 20 ± 0.8, G 5.5 mM & F 5.5 mM, 46 ± 5.8, p < 0.01). We also observed a 4-fold increase in extracellular oleate concentrations in the fructose-treated cells in keeping with increased hepatic cell secretion of these newly synthesized fatty acids. (figure 1b) (Mean ± SD extracellular oleate: G 5.5 mM, 10 ± 3; G 11.1 mM, 9.5 ± 0.8; G 5.5 mM & F 5.5 mM, 39 ± 7, p < 0.01). Oil-red-O staining (figure 1c & d) was increased in the hepatocytes co-treated with 11 mM glucose plus 0.55 mM fructose in comparison to 11.1 mM glucose-treatment alone (** p < 0.01) reflecting higher triglyceride content. To assess the contribution of the sugars to de novo fatty acid synthesis, we also examined tracer substrate-derived acetyl-CoA enrichment in cell palmitate. Acetyl-CoA contribution to new palmitate synthesis from glucose (figure 1e) was relatively low at 1.88% and was further decreased by fructose-treatment, likely due to the direct contribution of fructose derived acetyl-CoA to palmitate synthesis.

### Fructose impairs glucose-induced hepatic triglyceride synthesis and hydrolysis

As our metabolomic studies had demonstrated increased de novo triglyceride synthesis and secretion in hepatic cells cultured in 11 mM glucose co-treated with low concentration fructose, we next sought to explore the mechanism(s) by which fructose resulted in increased hepatic triglycerides. Hepatic triglyceride synthesis involves multiple metabolic pathways, including glycolysis and pyruvate oxidation which generate acetyl-CoA. Acetyl CoA is then converted to malonyl-CoA by the enzyme acetyl CoA carboxylase before conversion to palmitate by fatty acid synthase [26].

**Figure 2:**
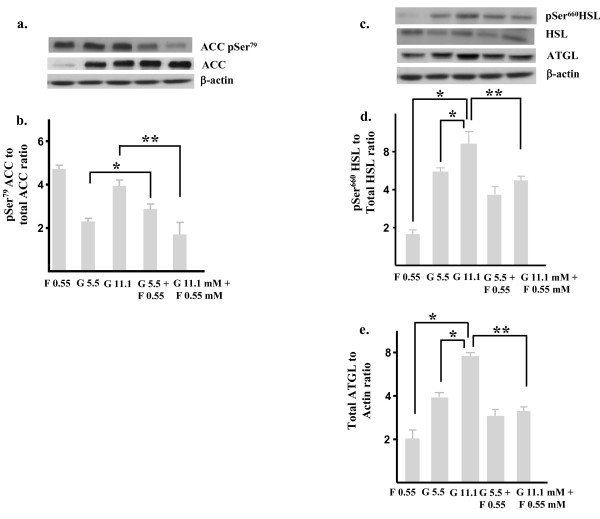
**Fructose impairs glucose-induced hepatic triglyceride synthesis and hydrolysis**. Immunoblots and quantitation depicting (a & b) pSer^79 ^Acetyl CoA Carboxylase (ACC), total ACC and (c & d) pSer^660 ^hormone sensitive lipase (HSL), total HSL and (c & e) total adipose triglyceride lipase (ATGL) in human HepG2 cells following incubation (72h) in glucose (5.5 & 11.1 mM) or fructose (0.55 mM) alone or an admixture of glucose (5.5 & 11.1 mM) together with 0.55 mM fructose concentrations. β-actin served as a loading control. G; glucose; F, fructose. * p < 0.05, ** p < 0.01.

Therefore, we first compared effects of glucose- (5.5 and 11.1 mM) or fructose- (0.55 mM) treatment alone *in vitro *on several key enzymes involved in hepatic triglyceride synthesis and hydrolysis in human hepatic HepG2 cells and primary murine hepatic cell cultures. As depicted in figure 2a-b, *in vitro *glucose-treatment (5.5 & 11.1 mM) increased serine-phosphorylated acetyl Co-carboxylase (pSer^79 ^ACC) levels, in keeping with increased de novo fatty acid synthesis. In contrast, following fructose-treatment alone (0.55 mM) although total ACC levels fell, corrected pSer^79 ^ACC expression was similar to that observed in glucose-treated cells. However, when fructose (0.55 mM) was added to either 5.5 or 11.1 mM glucose, pSer^79 ^ACC levels were lower in comparison to levels observed in glucose-treated cells (* p < 0.05, ** p < 0.01). We also compared expression of activated serine-phosphorylated hormone sensitive lipase (pSer^660 ^HSL) and adipose triglyceride lipase (ATGL) expression in the HepG2 cells. As demonstrated in figure 2c-e, pSer^660 ^HSL expression also increased in HepG2 cells treated with glucose (p = ns) but was significantly lower in cells treated with fructose only (* p < 0.05) and combination fructose- and glucose-treatment resulted in lower pSer^660 ^HSL expression compared to glucose alone (*, p < 0.01). ATGL expression was higher in glucose versus fructose-treated cells and combination treatment led to reduced ATGL expression (figure 2e) (* p < 0.05).

### Fructose impairs murine hepatic triglyceride synthesis *in vitro*

**Figure 3:**
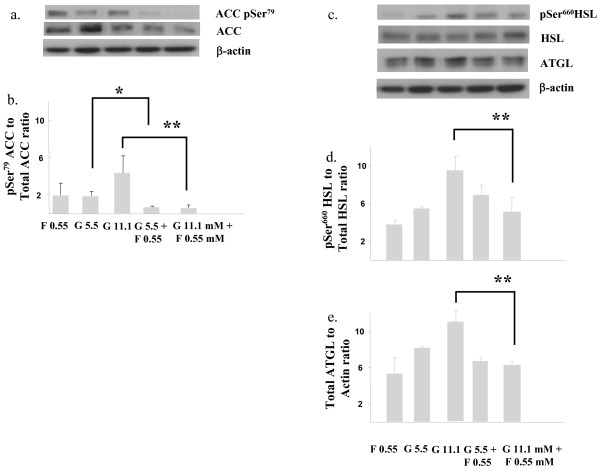
**Fructose impairs ACC phosphorylation in primary murine hepatocytes**. Immunoblots and quantitation depicting (a & b) pSer^79 ^Acetyl CoA Carboxylase (ACC) and total ACC and (c & d) pSer^660 ^hormone sensitive lipase (HSL), total HSL and (c & e) total adipose triglyceride lipase (ATGL) in primary cultures of murine hepatocytes following incubation (72h) in glucose (5.5 & 11.1 mM) or fructose (0.55 mM) alone or an admixture of glucose (0.055 & 11.1 mM) together with 0.55 mM fructose concentrations. β-actin served as a loading control. G; glucose; F, fructose. * p < 0.05, ** p < 0.01.

We also tested the effects of combination fructose- and glucose-treatment in primary murine hepatocytes. As depicted in figure 3a & b, addition of 5.5 mM glucose or fructose alone to primary murine hepatocytes resulted in similar corrected pSer^79 ^ACC levels. In contrast, addition of fructose to the primary hepatocytes treated with 5.5 mM glucose led to a 1.5-fold reduction in pSer^79 ^ACC in comparison to 5.5 mM glucose-treatment alone (*, p < 0.05). The effect of co-treatment with fructose (0.55 mM) and 11.1 mM glucose was even more striking and resulted in virtual abrogation of pSer^79 ^ACC in the primary hepatocytes with a ~5-fold reduction in pSer^79 ^ACC levels in comparison to 11.1 mM glucose-treatment alone (** p < 0.01). As demonstrated in figure 3c-e, co-treatment with fructose and glucose alone suppressed 11 mM glucose-stimulated p^Ser ^660HSL (p < 0.01) and ATGL levels (p < 0.01) in the primary murine hepatocytes in comparison to 11.1 mM glucose-treatment alone.

### Dietary fructose impairs glucose disposal and glucose-stimulated insulin release

**Figure 4:**
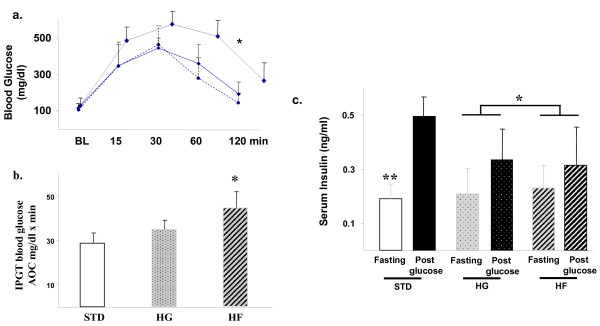
**Effect of dietary fructose on glucose disposal**. (a) Intraperitoneal glucose tolerance tests in mice following 12-week standard (STD) (*hatched line*) high glucose (HG) (*solid line*) or high fructose (HF) (*dotted line*) diets depicting glucose levels after overnight fast at baseline (0), 15, 30, 60 and 120 minutes after glucose 1 g/kg administration. The area under the curve (AOC) (Mean SD) for each of the groups is depicted in the bar chart (d). (e) Insulin levels at baseline and following the IP glucose load in the three groups. STD, standard diet; HG, high glucose diet; HF, high fructose diet. *, p < 0.05, **, p < 0.01.

In light of our *in vitro *findings, we next examined effects of dietary fructose versus glucose on hepatic triglyceriude synthesis *in vivo*. Although fructose-feeding has been shown to induce impaired insulin action and increased hepatic steatosis in several rodent species including rats and hamsters, C57Bl/6 mice appear comparatively resistant to carbohydrate-induced effects. Therefore, we fed athymic Nu/Nu mice either isocaloric defined diets, so-called standard (STD-), 60% glucose (HG-) or 60% fructose (HF-) diets for 12 weeks and then measured glucose and insulin levels across a standard intraperitoneal glucose tolerance test (1 gm/kg glucose). As depicted in figure 4a-d, glucose disposal as measured by calculating the area under the glucose curve, was similar in mice fed either the HG- and STD-diets but reduced in the fructose fed animals (*, p < 0.05 STD or HG vs HF). In STD-diet fed mice, insulin levels rose ~2.6-fold from baseline following the glucose load, p < 0.002 (figure 4e), whereas mice fed the HG- diet demonstrated a reduced ~1.6-fold increase in insulin levels. Fructose-fed mice exhibited an even lower 1.3-fold glucose-stimulated increase in insulin (fructose versus glucose, p < 0.05). The results demonstrate that both high glucose- and high fructose-feeding resulted in reduced glucose-stimulated insulin release in comparison to standard diet in the athymic Nu/Nu mouse model and that fructose-fed mice also exhibited impaired glucose disposal.

### Refined carbohydrate induces hepatic steatosis and elevated hepatic triglyceride levels *in vivo*

**Figure 5:**
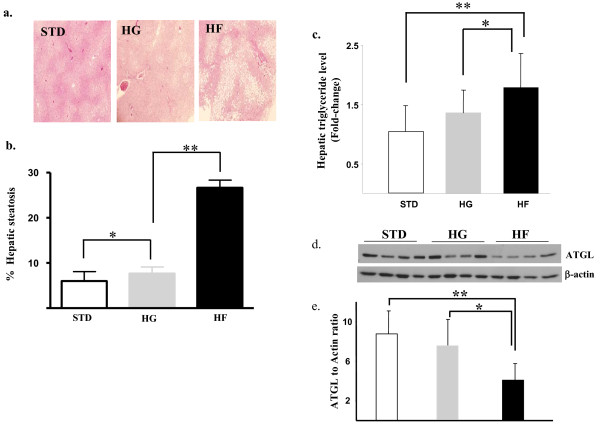
**Fructose increases hepatic steatosis and triglyceride content**. (a) Photomicrographs depicting hematoxylin & eosin staining of hepatic tissues (x 200), (b) quantitation of hepatic steatosis and (c) triglyceride levels in livers harvested from mice fed the various diets. Immunoblot depiction and quantitation (d & e) of total ATGL expression in hepatic tissue protein lysates harvested from 12 mice following 12-week standard (STD), high glucose (HG) or high fructose (HF) diets. μ-actin served as a loading control. * p < 0.05, ** p < 0.01.

Food-intake and final body weights did not differ amongst the STD-, HG-, or HF- diet fed mice. Liver weights were higher in mice fed the HF-diet in comparison to the HG- and STD-diet fed mice (mean ± SEM hepatic Wt: STD; 0.9 ± 0.01; HG, 1.2 ± 0.01; HF 1.6 ± 0.01 g, p < 0.05 ). Hepatic histology demonstrated markedly increased micro and macro steatosis in fructose-fed mice which was 2.7-fold higher compared to HG-fed animals (p < 0.05) and 4.5-fold higher than steatosis observed in STD-diet fed mice (p < 0.01) (Ffigure 5a & b). Hepatic triglyceride levels were 1.3-fold higher in liver tissues from HG-fed mice in comparison to STD-fed animals but the highest hepatic Tg levels were seen in HF-fed mice. Hepatic Tg levels in fructose-fed mice were 1.7-fold higher than STD-fed (p < 0.01), and 1.3-fold higher than HG-fed mice (p < 0.05) (figure 5c), illustrating the potent effect of dietary fructose on hepatic triglyceride levels. Given our *in vitro *findings, we also examined expression of the hepatic triglyceride enzymes (pSer^79 ^ACC, total ACC, pSer^660 ^HSL, total HSL and ATGL) in a randomly selected subset of 12 mice fed the STD-, 60% glucose or 60%-fructose-containing diets. No significant alterations in *in vivo *pSer^660 ^HSL or pSer^79 ^ACC expression (not shown) were observed but as demonstrated in figure 5c & d, lower ATGL levels were observed in fructose-fed mice in comparison to glucose- (p < 0.05) or standard-diet animals (p < 0.01)..

## Discussion

Several human and animal studies have demonstrated that dietary fructose intake increases triglyceride levels [27-34]. In humans, these lipid alterations occur quite quickly as evidenced by a 10 week study in 32 men & women who ate a 15% protein, 30% fat and 55% carbohydrate diet where 25% of calories were replaced with a glucose- or fructose-containing drink. De novo hepatic lipogenesis, markers of altered lipid metabolism and lipoprotein remodeling, including fasting apoB, LDL, small dense LDL and oxidized LDL all increased [32]. The postprandial triglyceride response in particular increased 10% in the fructose-treated group in comparison to glucose, emphasizing the role of the hepatic response to fructose loads [32]. However, the molecular mechanisms by which high fructose diets induce abnormalities in liver triglyceride metabolism are not fully understood [35-37].

Synthesis of hepatic TGs involves multiple metabolic pathways, including glycolysis and pyruvate oxidation to generate acetyl-CoA. Our ^13^C glucose tracer studies demonstrate increased intracellular palmitate and extracellular oleate concentration in fructose and glucose-cotreated cells compared to glucose-treated cells in keeping with increased "de novo" fatty acid synthesis [26]. Our molecular *in vitro *studies demonstrate that addition of fructose to glucose-treated hepatic cells leads to reduced serine-phosphorylated ACC, HSL and ATGL [38]. Therefore, in addition to serving as a substrate for enhanced de-novo fatty acid synthesis, fructose and or its intermediate metabolites may also alter expression of several enzymes involved in hepatic lipid synthesis and hydrolysis pathways.

As we observed increased extracellular oleate concentrations in the fructose-treated liver cells, this study indicates that in addition to increasing de novo hepatic fatty acid synthesis, fructose-treatment also leads to enhanced triglyceride secretion, as when triglycerides are assembled for release, they require oleate to provide fluidity. The combination of increased fatty acid synthesis and triglyceride release provides novel insight into the hepatic steatosis that has been demonstrated in animal models following dietary fructose and in humans where fructose intake correlates with prevalence of fatty liver [27,28,34,39].

Our studies have focused on the liver as approximately 40% of ingested fructose is extracted and metabolized in the liver. Adipose tissue serves as the main TG store but they are also stored in non-adipose tissues including muscle and liver where they can provide fatty acid substrate for metabolic processes [40]. Additionally, studies have shown that fructose plays a specific role in the pathogenesis of hepatic steatosis and metabolic syndrome due to differential hepatic fructose metabolism. Unlike most tissues which contain only hexokinase that competitively phosphorylates glucose or fructose at the sixth carbon [41], hepatocytes also express fructokinase-1 that phosphorylates fructose on ^1^C to generate fructose 1- phosphate. Fructose-1-phosphate can then be metabolized into dihydroxyacetone phosphate and glyceraldehydes entering the latter steps of the Embden-Meyerhof pathway to generate triacylglycerol. As this entry point is distal to the glycolysis rate limiting enzyme phospho fructokinase-1, unlike glucose, fructose can serve as a relatively unregulated source of acetyl-CoA [4]. However, the contribution of glycolysis to de-novo hepatic fatty acid synthesis is small (< 10%) as supported by our metabolomic studies. Therefore, other mechanisms to increase fatty acid synthesis are likely more important including, fructose 1, 6 bis-phosphate which is a potent regulator of fatty acid synthesis by directly activating fatty acid synthase.

Although dietary carbohydrate induces insulin resistance in rats and hamsters, most commonly employed mice strains such as C57Bl/6 appear to be relatively resistant to CHO-induced metabolic syndrome [42]. The athymic Nu/Nu mouse represents a model of non obese non-immune non-insulin-dependent diabetes [43,44] and has been used to delineate immune-mediated pancreatic islet effects in insulin-dependent diabetes mellitus [45,46]. Our studies demonstrate that unlike the C57Bl/6 mouse, increased CHO feeding to Nu/Nu mice results in development of a metabolic syndrome phenotype with modestly impaired peripheral glucose disposal. In contrast to our *in vitro *finding of reduced pS79ACC levels following fructose, we did not see changes in *in vivo *hepatic pS79ACC, ACC and/or pSer^660 ^HSL or total HSL expression [39]. This is unusual, given that ACC is the rate-limiting enzyme in lipogenesis but may be due to other actions of the sugars to alter ACC aggregation or other longer chain CoA. Given that glucose-stimulated insulin levels were attenuated in the fructose-fed mice in comparison to glucose- and standard-diet fed mice, our studies also suggest that fructose-fed mice may exhibit a degree of beta-cell impairment and this is an area that we are currently investigating.

It is important to acknowledge that our animal *in vivo *studies employed higher refined carbohydrate content (60% of energy) than would be consumed in a typical western diet (7.5-20% of energy) and the relevance of *in vivo *findings in these stylized animal models to humans can reasonably be questioned. However, our *in vitro *study fructose concentrations (0.5 mM) are easily attainable in the peripheral human circulation and fructose concentrations can be predicted to be higher in post-prandial portal venous blood in subjects consuming average Western diets containing 10-15% refined carbohydrate [32].

It is widely appreciated that increases in triglycerides are typically more pronounced in obese patients with insulin resistance. Our studies demonstrating that the fructose effect to interfere with hepatic lipid metabolism is more marked in hyperglycemic conditions is of importance to diabetic patients as they suggest that fructose consumption may exacerbate an already existing adverse metabolic profile especially in diabetic patients who have poor glycemic control. However, the adverse effects of fructose on hepatic lipid metabolism are not restricted to poorly controlled diabetic patients as our *in vitro *experiments demonstrate that even low concentrations of fructose can disrupt normal hepatic glucose metabolism.

In summary, our studies provide novel insight into the mechanism of fructose-induced hepatic hypertriglyceridemia and show that fructose, a component of sucrose (table sugar) and high fructose corn syrup, leads to increased de novo hepatic fatty acid synthesis and release of triglycerides in comparison to glucose. This not only entails differential utilization of fructose as a substrate for de novo fatty acid synthesis but may also involve alterations in the levels of key enzymes involved with fatty acid synthesis and hydrolysis including ACC-, ATGL- and HSL. Furthermore, they support the contention that fructose-containing foods can have detrimental effects on hepatic lipid synthesis with potentially adverse consequences on cardiovascular risk. They have important clinical implications given the increase in obesity rates and its associated complications, including diabetes.

## Competing interests

The authors declare that they have no competing interests.

## Authors' contributions

DH conducted the in vitro and in vivo studies and drafted the manuscript, TD assisted with in vitro studies, SY participated in acquiring images, WHY assisted with histological interpretation, LGB conducted the metabolomic studies and APH conceived of the study, participated in its design and helped write the manuscript. All authors read and approved the final manuscript.

## References

[B1] Bayturan O Tuzcu EM Lavoie A Hu T Wolski K Schoenhagen P Kapadia S Nissen SE Nicholls SJ The metabolic syndrome, its component risk factors, and progression of coronary atherosclerosis Arch. Intern. Med 2010 170 478 84 10.1001/archinternmed.2009.551 20212186

[B2] Basciano H Federico L Adeli K Fructose, insulin resistance, and metabolic dyslipidemia Nutr. Metab 2005 2 1 14 10.1186/1743-7075-2-5 PMC552336 15723702

[B3] Hallfrisch J Metabolic effects of dietary fructose FASEB 1990 4 2652 2660 10.1096/fasebj.4.9.2189777 2189777

[B4] Mayes PA Intermediary metabolism of fructose Am. J. Clin. Nutr 1993 58 Supp 5 10.1093/ajcn/58.5.754S 8213607

[B5] Tetri LH Basaranoglu M Brunt EM Yerian LM Neuschwander Tetri BA Severe NAFLD with hepatic necroinflammatory changes in mice fed trans fats and a high-fructose corn syrup equivalent Am. J. Physiol. Gastrointest. Liver Physiol 2008 295 G987 G995 10.1152/ajpgi.90272.2008 18772365 PMC4059366

[B6] Rutledge AC Adeli K Fructose and metabolic syndrome: Pathophysiology and molecular mechanisms Nutrition Rev 2007 65 S13 23 10.1301/nr.2007.jun.S13-S23 17605309

[B7] Ginsburg GS Safran C Pasternak RC Frequency of low serum high-density lipoprotein cholesterol levels in hospitalized patients with "desirable"total cholesterol levels Am. J. Cardiol 1991 1 187 92 10.1016/0002-9149(91)90742-4 2063780

[B8] Abbot RD Wilson PWF Kannel WB Castelli WP High density lipoprotein cholesterol, total cholesterol screening, and myocardial infarction. The Framingham Heart Study Arteriosclerosis 1988 8 207 11 3370018 10.1161/01.atv.8.3.207

[B9] Castelli WP Doyle JT Gordon T Hames CG Hjortland MC Hulley SB Kagan A Zukel WJ HDL cholesterol and other N lipids in coronary hean disease: the cooperative lipoprotein pheno type study Circulation 1977 55 767 72 191215 10.1161/01.cir.55.5.767

[B10] Reckless JPD Betteridge DJ Wu P Payne B Galton DJ High-density and low density lipoproteins and prevalence of vascular disease in diabetesmellitus Br. Med. J 1978 1 883 6 10.1136/bmj.1.6117.883 205308 PMC1603755

[B11] Havel RJ High density lipoproteins, cholesterol transport and coronary heart disease Circulation 1979 60 1 3 221132 10.1161/01.cir.60.1.1

[B12] Nestle PJ Readon M Billington T In vivo transport of cholesterol esters from high density lipoproteins to very low density lipoproteins in man Biochim. Biophys. Acta 1979 573 403 7 221036 10.1016/0005-2760(79)90073-0

[B13] Carlson LA Bolliger LE Ahfeldt PE Risk factors for myocardial infarction in the Stockholm perspective study: a 14-year follow-up focusing on the role of plasma triglyceride and cholesterol Acta. N. Med. Scand 1979 706 351 525434

[B14] Fontbonne A Eschwège E Cambien F Richard JL Ducimetière P Thibult N Warnet JM Claude JR Rosselin GE Hypertriglyceridemia as a risk factor of coronary heart disease mortality in subjects with impaired glucose tolerance or diabetes: results from the II-year follow-up of the Paris Prospective Study Diabetologia 1989 32 34 10.1007/BF00265546 2666216

[B15] West KM Ahuja MM Bennett PH Czyzyk A De Acosta OM Fuller JH Grab B Grabauskas V Jarrett RJ Kosaka K The role of circulating glucose and triglyceride concentrations and their interaction with other "risk factors" as determinants of anerial disease in nine diabetic populations: samples from the WHO multinational study Diabetes Care 1983 6 361 9 10.2337/diacare.6.4.361 6617413

[B16] Hollenbeck CB Dietary fructose effects on lipoprotein metabolism and risk for coronary artery disease Am. J. Clin. Nutr 1993 58 800S 809S 8213613 10.1093/ajcn/58.5.800S

[B17] Reaven GM Lerner RL Stem MP Farquhar JW Role of insulin in endogenous hypenriglyceridemia J. Clin. Invest 1967 46 175 7 10.1172/JCI105666 PMC292926 6061748

[B18] Olefsky JM Farquhar JW Reaven GM Reappraisal of the role of insulin hypenriglyceridemia Am. J. Med 1974 57 551 10.1016/0002-9343(74)90006-0 4372881

[B19] Tobey TA Greenfield MA Kraemer F Reaven GM Relationship between insulin resistance, insulin secretion, very low density lipoprotein kinetics and plasma triglyceride levels in normotriglyceridemic men Metabolism 1981 30 165 7I 10.1016/0026-0495(81)90167-0 7007804

[B20] Lavaroni I Sanders S Scoll S Reaven GM Effect of fructose feeding on insulin secretion and insulin action in the rat Metabolism 1980 29 970 3 10.1016/0026-0495(80)90041-4 6999292

[B21] Storlien LH Kraegan EW Jenkins AD Chisholm OJ Effects of sucrose vs starch diets on in vivo insulin action, thermogenesis, and obesity in rats Am. J. Clin. Nutr 1988 47 420 7 3348154 10.1093/ajcn/47.3.420

[B22] Thorburn AW Storlien LH Jenkins AD Fructose-induced in vivo insulin resistance and elevated plasma triglyceride levels in rats Am. J. Clin. Nutr 1989 49 1155 63 2658534 10.1093/ajcn/49.6.1155

[B23] Davis RC Castellani LW Hosseini M Ben-Zeev O Mao HZ Weinstein MM Jung DY Jun JY Kim JK Lusis AJ Péterfy M Early hepatic insulin resistance precedes the onset of diabetes in obese C57BLKS-db/db mice Diabetes 2010 59 1616 25 10.2337/db09-0878 20393148 PMC2889760

[B24] Cascante M Boros LG Comin B Atauri P Centelles JJ Lee W-NP Metabolic control analysis in drug discovery and disease Nature Biotechnology 2002 20 246 249 10.1038/nbt0302-243 11875424

[B25] Hwang JT Park IJ Shin JI Lee YK Lee SK Baik HW Ha J Park OJ Genistein, EGCG, and capsaicin inhibit adipocyte differentiation process via activating AMP-activated protein kinase Biochem. Biophys. Res. Commun 2005 338 694 699 10.1016/j.bbrc.2005.09.195 16236247

[B26] Postic C Girard J Contribution of de novo fatty acid synthesis to hepatic steatosis and insulin resistance: lessons from genetically engineered mice J. Clin. Invest 2008 118 829 10.1172/JCI34275 18317565 PMC2254980

[B27] Faeh D Minehira K Schwarz JM Periasamy R Park S Tappy L Effect of fructose overfeeding and fish oil administration on hepatic de novo lipogenesis and insulin sensitivity in healthy men Diabetes 2005 54 1907 1913 10.2337/diabetes.54.7.1907 15983189

[B28] Lê KA Faeh D Stettler R Ith M Kreis R Vermathen P Boesch C Ravussin E Tappy L A 4-wk high-fructose diet alters lipid metabolism without affecting insulin sensitivity or ectopic lipids in healthy humans Am. J. Clin. Nutr 2006 84 1374 1379 17158419 10.1093/ajcn/84.6.1374

[B29] Abdel-Sayed A Binnert C Lê KA Bortolotti M Schneiter P Tappy L A high-fructose diet impairs basal and stress-mediated lipid metabolism in healthy male subjects Br. J. Nutr 2008 100 393 399 10.1017/S000711450789547X 18205992

[B30] Teff KL Elliott SS Tschöp M Kieffer TJ Rader D Heiman M Townsend RR Keim NL D'Alessio D Havel PJ Dietary fructose reduces circulating insulin and leptin, attenuates postprandial suppression of ghrelin, and increases triglycerides in women J. Clin. Endocrinol. Metab 2004 89 2963 72 10.1210/jc.2003-031855 15181085

[B31] Swarbrick MM Stanhope KL Elliott SS Graham JL Krauss RM Christiansen MP Griffen SC Keim NL Havel PJ Consumption of fructose sweetened beverages for 10 weeks increases postprandial triacylglycerol and apolipoprotein-B concentrations in overweight and obese women Br. J. Nutr 2008 100 947 52 10.1017/S0007114508968252 18384705 PMC3038917

[B32] Stanhope KL Schwarz JM Keim NL Griffen SC Bremer AA Graham JL Hatcher B Cox CL Dyachenko A Zhang W McGahan JP Seibert A Krauss RM Chiu S Schaefer EJ Ai M Otokozawa S Nakajima K Nakano T Beysen C Hellerstein MK Berglund L Havel PJ Effects of consuming fructose- or glucose-sweetened beverages for 10 weeks on lipids, insulin sensitivity and adiposity J. Clin. Invest 2009 119 1322 1334 10.1172/JCI37385 19381015 PMC2673878

[B33] Lê KA Ith M Kreis R Faeh D Bortolotti M Tran C Boesch C Tappy L Fructose overconsumption causes dyslipidemia and ectopic lipid deposition in healthy subjects with and without a family history of type 2 diabetes Am. J. Clin. Nutr 2009 89 1760 1765 19403641 10.3945/ajcn.2008.27336

[B34] Teff KL Grudziak J Townsend RR Dunn TN Grant RW Adams SH Keim NL Cummings BP Stanhope KL Havel PJ Endocrine and metabolic effects of consuming fructose- and glucose-sweetened beverages with meals in obese men and women: influence of insulin resistance on plasma triglyceride responses J. Clin. Endo. Metab 2009 94 1562 1569 10.1210/jc.2008-2192 PMC2684484 19208729

[B35] Schaffer JE Lipotoxicity: when tissues overeat Curr. Opin. Lipidol 2003 14 281 287 10.1097/00041433-200306000-00008 12840659

[B36] Unger RH Minireview: Weapons of lean body mass destruction: the role of ectopic lipids in the metabolic syndrome Endocrinology 2003 144 5159 5165 10.1210/en.2003-0870 12960011

[B37] Van Herpen NA Schrauwen-Hinderling VB Lipid accumulation in non-adipose tissue and lipotoxicity Physiol. Behav 2008 94 231 241 10.1016/j.physbeh.2007.11.049 18222498

[B38] Zimmermann R Strauss JG Haemmerle G Schoiswohl G Birner-Gruenberger R Riederer M Lass A Neuberger G Eisenhaber F Hermetter A Zechner R Fat mobilizationin adipose tissue is promoted by adipose triglyceride lipase Science 2004 306 1383 6 10.1126/science.1100747 15550674

[B39] Watt MJ Storing up trouble: does intramyocellular trigelyceride accumulation protect skeletal muscle form insulin resistance? Proc. Aust. Physiol. Soc 2008 39 149 57 10.1111/j.1440-1681.2008.05075.x 18986321

[B40] Van Schaftingen E Glycolysis revisited Diabetologia 1993 36 581 8 10.1007/BF00404065 8395434

[B41] Dirlewanger M Schneiter P Jéquier E Tappy L Effects of fructose on hepatic glucose metabolism in humans Am. J. Physiol. Endocrinol. Metab 2000 279 E907 11 11001775 10.1152/ajpendo.2000.279.4.E907

[B42] Zeidler A Tosco C Kumar D Slavin B Parker J Spontaneous hyperglycemia and impaired glucose tolerance in athymic nude BALB/c mice Diabetes 1982 31 821 5 10.2337/diabetes.31.9.821 6761217

[B43] Zeidler A Kumar D Johnson C Parker J Development of a diabetes-like syndrome in an athymic nude Balb/c mouse colony Exp. Cell Biol 1984 52 145 9 6365646

[B44] Buschard K The thymus-dependent immune system in the pathogenesis of type 1 (insulin-dependent) diabetes mellitus. Animal model and human studies Danish Med. Bull 1985 32 139 51 3160550

[B45] Tuch BE Ng AB Jones A Turtle JR Histologic differentiation of human fetal pancreatic explants transplanted into nude mice Diabetes 1984 33 1180 7 10.2337/diabetes.33.12.1180 6149970

[B46] Herzberg GR Rogerson M Interaction of the level of dietary fat and type of carbohydrate in the regulation of hepatic liopgenesis in the mouse Can. J. Physiol. Pharmacol 1982 60 912 9 6127153 10.1139/y82-128

